# Phase III study of bilayer sustained-release tramadol tablets in patients with cancer pain: a double-blind parallel-group, non-inferiority study with immediate-release tramadol capsules as an active comparator

**DOI:** 10.1007/s00520-023-08242-z

**Published:** 2023-12-29

**Authors:** Masaharu Shinkai, Noriyuki Katsumata, Shinichi Kawai, Shoichi Kuyama, Osamu Sasaki, Yasuhiro Yanagita, Minoru Yoshida, Shima Uneda, Yasushi Tsuji, Hidenori Harada, Yasunori Nishida, Yasuhiro Sakamoto, Daisuke Himeji, Hitoshi Arioka, Kazuhiro Sato, Ryo Katsuki, Hiroki Shomura, Hideshi Nakano, Hideaki Ohtani, Kazutaka Sasaki, Takeshi Adachi

**Affiliations:** 1 Tokyo Shinagawa Hospital, Tokyo, Japan; 2 Nippon Medical School Musashikosugi Hospital, Kanagawa, Japan; 3 Toho University, Tokyo, Japan; 4 National Hospital Organization Iwakuni Clinical Center, Yamaguchi, Japan; 5 Miyagi Cancer Center, Miyagi, Japan; 6 Gunma Prefectural Cancer Center, Gunma, Japan; 7 Japanese Red Cross Kumamoto Hospital, Kumamoto, Japan; 8 Tonan Hospital, Hokkaido, Japan; 9 Yamaguchi University Hospital, Yamaguchi, Japan; 10 Keiyukai Sapporo Hospital, Hokkaido, Japan; 11 Osaki Citizen Hospital, Miyagi, Japan; 12 Miyazaki Prefectural Miyazaki Hospital, Miyazaki, Japan; 13 Yokohama Rosai Hospital, Kanagawa, Japan; 14 Nagaoka Red Cross Hospital, Niigata, Japan; 15 National Hospital Organization Ureshino Medical Center, Saga, Japan; 16 Japan Community Health Care Organization Hokkaido Hospital, Hokkaido, Japan; 17 Department of Clinical Development, Nippon Zoki Pharmaceutical Co., Ltd., Osaka, Japan

**Keywords:** Cancer pain, Tramadol, Sustained-release, Bilayer tablets, Randomized controlled study, Non-inferiority study

## Abstract

**Purpose:**

We investigated whether twice-daily administration of a bilayer tablet formulation of tramadol (35% immediate-release [IR] and 65% sustained-release) is as effective as four-times-daily IR tramadol capsules for managing cancer pain.

**Methods:**

This randomized, double-blind, double-dummy, active-comparator, non-inferiority study enrolled opioid-naïve patients using non-steroidal anti-inflammatory drugs or acetaminophen (paracetamol) to manage cancer pain and self-reported pain (mean value over 3 days ≥ 25 mm on a 100-mm visual analog scale [VAS]). Patients were randomized to either bilayer tablets or IR capsules for 14 days. The starting dose was 100 mg/day and could be escalated to 300 mg/day. The primary endpoint was the change in VAS (averaged over 3 days) for pain at rest from baseline to end of treatment/discontinuation.

**Results:**

Overall, 251 patients were randomized. The baseline mean VAS at rest was 47.67 mm (range: 25.6–82.7 mm). In the full analysis set, the adjusted mean change in VAS was − 22.07 and − 19.08 mm in the bilayer tablet (n = 124) and IR capsule (n = 120) groups, respectively. The adjusted mean difference was − 2.99 mm (95% confidence interval [CI] − 7.96 to 1.99 mm). The upper 95% CI was less than the predefined non-inferiority margin of 7.5 mm. Other efficacy outcomes were similar in both groups. Adverse events were reported for 97/126 (77.0%) and 101/125 (80.8%) patients in the bilayer tablet and IR capsule groups, respectively.

**Conclusion:**

Twice-daily administration of bilayer tramadol tablets was as effective as four-times-daily administration of IR capsules regarding the improvement in pain VAS, with comparable safety outcomes.

**Clinical trial registration:**

JapicCTI-184143/jRCT2080224082 (October 5, 2018).

**Supplementary Information:**

The online version contains supplementary material available at 10.1007/s00520-023-08242-z.

## Introduction

Pain is a common symptom in cancer patients; some studies suggested that almost half of patients experience pain at least 3 months after completing curative treatment, and nearly a third experience moderate to severe pain [1, 2]. Cancer pain may be caused by the cancer itself or metastases, or may be related to the treatments (e.g., surgical pain, neuropathic pain after chemotherapy) [3]. Despite its high prevalence and significant impact on patient well-being, it was reported that cancer pain is under-treated in approximately one-third of patients [3], constituting an important unmet need in clinical practice.

Clinical guidelines for managing cancer pain, including those developed by the World Health Organization (WHO) [4], National Comprehensive Cancer Network [5], American Society of Clinical Oncology [6], European Society of Medical Oncology [7], and the Japanese Society for Palliative Medicine [8], suggest that pain should be managed according to the patient’s pain intensity, and that treatment may include an opioid, such as tramadol. In particular, the WHO guidelines position opioids as drugs that should be used according to the clinical assessment and pain intensity for rapid, effective, and safe pain management from the initiation of pain management, even if not based on the conventional three-step analgesia ladder. The guidelines also state that any opioid may be selected for cancer-related pain. Patients may require stronger opioids, other analgesics, or adjuvant therapies, the choice of which will depend on their clinical condition [4–8].

Tramadol is a weak μ-opioid receptor agonist that also inhibits norepinephrine and serotonin reuptake, with proven efficacy for managing chronic pain. Oral administration is preferred, with a regular dosing frequency every 4 or 12 h depending on the formulation prescribed (e.g., immediate-release or extended-release formulations). However, another administration route may be required in some patients.

With a view to improving the pharmacokinetic profile of orally administered tramadol, Nippon Zoki developed a new tramadol formulation as bilayer sustained-release (SR) tablets (hereafter bilayer tablets) in which the top layer comprises 35% of the dose as an immediate-release (IR) formulation and the lower layer comprises 65% of the dose as a SR formulation administered twice-daily (Twotram^®^ tablets; Nippon Zoki Pharmaceutical Co., Ltd.) [9, 10]. This is the first twice-daily tramadol formulation to be developed and marketed in Japan [10]. To date, several Phase III clinical studies have demonstrated the efficacy of these bilayer tablets for managing chronic non-cancer pain associated with knee osteoarthritis [11] and postherpetic neuralgia [12], and the long-term efficacy and safety were demonstrated in a 52-week study [10]. To expand the potential indications for the bilayer tablet, we performed a randomized controlled study to examine its effectiveness in Japanese patients with cancer pain by testing its non-inferiority versus IR tramadol capsules as an active comparator.

## Methods

The study was registered on the Japan Pharmaceutical Information Center clinical trial information (JapicCTI-184143) and Japan Registry of Clinical Trials (jRCT2080224082) (date registered: October 5, 2018).

### Patients

Patients were eligible for this study if they had been diagnosed with cancer, had an estimated survival of ≥ 3 months from the start of study drug administration, were currently using non-opioid analgesics (nonsteroidal anti-inflammatory drugs [NSAIDs] or acetaminophen [paracetamol]), had not previously used an opioid analgesic, and the physician deemed it necessary to start tramadol to manage cancer pain. Patients used a 100-mm visual analog scale (VAS) to assess their pain at rest on study days − 2, − 1, and 1 (where study day 1 was the day of starting treatment); only patients with a score of ≥ 25 mm averaged over the 3 days were eligible for the study. Other eligibility criteria included patients treated in an inpatient or outpatient setting, age ≥ 20 years, and adequate liver and renal functions. The major exclusion criteria are listed in the Supplementary Methods.

### Study design

**Fig. 1:**
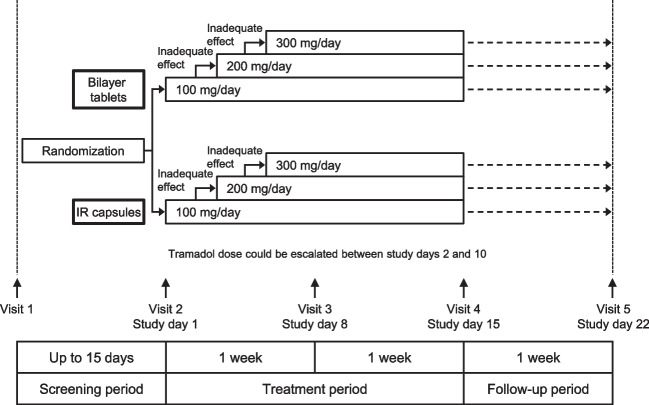
Study design. The patient’s eligibility was checked during the screening period (up to 15 days). Patients used a 100-mm visual analog scale (VAS) to assess their pain at rest on study days − 2, − 1, and 1 (where study day 1 was the day of starting treatment); only patients with a score of ≥ 25 mm averaged over the 3 days were eligible. The dosing procedure in each group is described in more detail in the Supplementary Methods. Patients received a dose of 50 mg/day in the evening on study day 1, followed by 100 mg/day on study day 2. On study days 2–10, the tramadol dose could be escalated by 100 mg/day to a maximum of 300 mg/day. The dose could not be escalated between study days 11 and 14. Rescue medication was permitted throughout the treatment period. *IR* immediate-release

This was a randomized, double-blind, double-dummy, active-comparator non-inferiority study comprising three periods: screening period, treatment period, and follow-up period (Fig. 1). In the treatment period with a double-dummy procedure, the patients took bilayer tablets (active drug or placebo) twice daily (morning and evening) and IR capsules (placebo or active drug) four times per day (morning, noon, evening, and before bed) according to the random allocation method in a blinded manner for up to 14 days (study days 1–15). The rationale for the 14-day treatment period is described in the Supplementary Methods. The dosing of study drugs and use of rescue medication are summarized in Fig. 1 and described in detail in the Supplementary Methods. Patients were randomized centrally using a dynamic allocation method in which study site and the patient’s mean score for VAS at rest (averaged over study days − 2, − 1, and 1) before the start of study drug administration were used as the allocation factors. After the 14-day treatment period, the patients entered a 7-day follow-up period, during which they could be prescribed tramadol, NSAIDs, or acetaminophen at the discretion of the investigator/subinvestigator. Approved and prohibited therapies are summarized in the Supplementary Methods. The investigator/subinvestigator at each study site was responsible for enrolling the patients using a web-based registration system. The allocation manager was responsible for assigning the study drugs, maintaining blinding, and storing the blinding code. Blinding was maintained until the database was locked.

### Endpoints

Every evening, just before administering the study drug, the patients evaluated their pain at rest and during movement over the previous 24-h period using a 100-mm VAS. This information was used to determine the primary endpoint—the change in the VAS for pain at rest from baseline (averaged over the 3 days before starting treatment) to the end of treatment (EOT; averaged over study days 12–14) or at discontinuation (averaged over the 3 days before discontinuation). A clinically relevant change in the VAS for pain was defined as a moderate or greater improvement during treatment relative to the baseline score using the chart shown in Supplementary Table 1; this definition was developed and utilized in prior studies in Japan [13, 14]. The Supplementary Methods describes the secondary endpoints and safety assessments. There were no changes to the study design after the first patient had been enrolled.

### Statistical analyses

In consideration of the sample size calculation (Supplementary Methods), it was planned to enroll 120 patients per group.

For this study, we defined three analysis populations. The full analysis set (FAS) comprised all patients who received at least one dose of study drug and for whom the primary endpoint (i.e., change in pain VAS at rest from baseline to the EOT or discontinuation) could be calculated for modified intention-to-treat analyses. The per-protocol set (PPS) comprised all patients in the FAS, excluding those with major protocol deviations (e.g., eligibility criteria, randomization/blinding violations, or non-compliance with study drug administration). The safety analysis set (SAF) comprised all patients who received at least one dose of the study drug.

The primary endpoint was analyzed using the FAS and verified using the PPS by analysis of covariance with treatment group as a fixed factor and the baseline VAS score as a covariate to estimate the adjusted mean change in each group and the between-group difference in adjusted mean change with 95% confidence intervals (CI). Non-inferiority was established if the upper limit of the CI for the between-group difference did not exceed the non-inferiority margin (7.5 mm). Descriptive statistics were also calculated for VAS scores at each visit. Other analyses are described in the Supplementary Methods. SAS version 9.4 (SAS Institute, Cary, NC, USA) was used for all data analyses.

## Results

### Patients

**Fig. 2:**
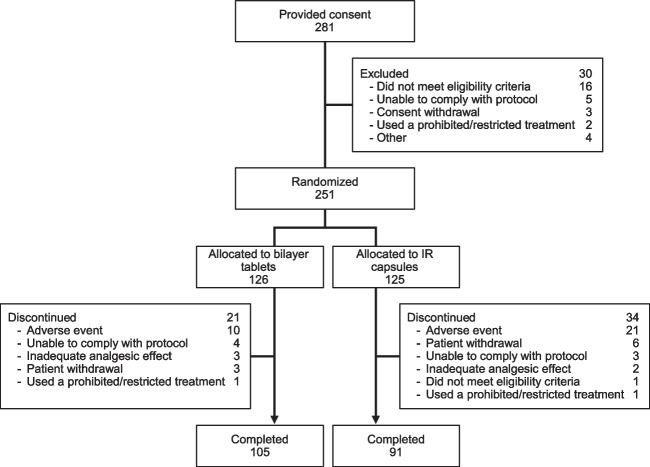
Patient disposition. A total of 251 patients were randomized to either the bilayer tablet or IR capsule groups. All of these patients received the allocated drugs, as randomized, and were included in the safety analysis set. Twenty-one patients discontinued treatment in the bilayer tablet group and 34 discontinued treatment in the IR capsule group. The most common reason for discontinuation was an adverse event, accounting for 10 patients in the bilayer tablet group and 21 patients in the IR capsule group. *IR* immediate-release

A total of 281 patients initially provided consent, of which 251 were randomized (126 to the bilayer tablet group and 125 to the IR capsule group) (Fig. 2). Of these, 105 completed the study in the bilayer tablet group and 91 in the IR capsule group (Fig. 2).

The baseline characteristics of patients in both groups (SAF) were similar (Table 1). At baseline, patients in both groups typically reported moderate–high levels of pain, with a mean VAS at rest of 47.67 mm, ranging from 25.6 to 82.7 mm. Most patients (81.7%) were treated as outpatients. The most common cancer site was the gastrointestinal tract (37.1%) followed by the bile duct/liver/pancreas (20.3%). The most common metastatic sites were bone (36.7%), liver (32.3%), and lower lymph nodes (29.1%). The main site of pain was the abdomen (43.4%) followed by the dorsal region (29.5%) and low back (23.9%). All of the patients were using concomitant drugs, including non-opioid analgesics in 98.8% and NSAIDs in 76.1%. Anti-cancer drugs were used in 68.9% of patients.

**Table 1:** Patient characteristics (safety analysis set)

		Bilayer tablets	IR capsules	Total
*N*		126	125	251
Age (year)	Mean ± SD	68.8 ± 9.4	68.2 ± 10.2	68.5 ± 9.8
Age category	< 65 years	36 (28.6)	41 (32.8)	77 (30.7)
	65–74 years	50 (39.7)	51 (40.8)	101 (40.2)
	≥ 75 years	40 (31.7)	33 (26.4)	73 (29.1)
Sex	Male	76 (60.3)	70 (56.0)	146 (58.2)
	Female	50 (39.7)	55 (44.0)	105 (41.8)
Mean VAS at rest (mm)	Mean ± SD	48.21 ± 16.09	47.12 ± 14.86	47.67 ± 15.47
	Range	26.1–82.7	25.6–81.8	25.6–82.7
	25 to < 45 mm	61 (48.4)	62 (49.6)	123 (49.0)
	≥ 45 mm	65 (51.6)	63 (50.4)	128 (51.0)
Status	Outpatient	102 (81.0)	103 (82.4)	205 (81.7)
	Inpatient	24 (19.0)	22 (17.6)	46 (18.3)
Tumor type/location^a^	Gastrointestinal	46 (36.5)	47 (37.6)	93 (37.1)
	Bile duct/liver/pancreas	22 (17.5)	29 (23.2)	51 (20.3)
	Genitourinary or reproductive organ	26 (20.6)	14 (11.2)	40 (15.9)
	Lung	16 (12.7)	20 (16.0)	36 (14.3)
	Breast	13 (10.3)	15 (12.0)	28 (11.2)
	Head or neck	0	2 (1.6)	2 (0.8)
	Others	7 (5.6)	5 (4.0)	12 (4.8)
Metastatic site^a^	Bone	51 (40.5)	41 (32.8)	92 (36.7)
	Liver	44 (34.9)	37 (29.6)	81 (32.3)
	Lower lymph nodes	40 (31.7)	33 (26.4)	73 (29.1)
	Lung	35 (27.8)	36 (28.8)	71 (28.3)
	Upper lymph nodes	27 (21.4)	25 (20.0)	52 (20.7)
	Ascites	10 (7.9)	12 (9.6)	22 (8.8)
	Pleural effusion	11 (8.7)	10 (8.0)	21 (8.4)
	Brain	8 (6.3)	2 (1.6)	10 (4.0)
	Skin	5 (4.0)	4 (3.2)	9 (3.6)
	Others	47 (37.3)	35 (28.0)	82 (32.7)
Site of pain^a^	Abdomen	57 (45.2)	52 (41.6)	109 (43.4)
	Dorsal	35 (27.8)	39 (31.2)	74 (29.5)
	Low back	33 (26.2)	27 (21.6)	60 (23.9)
	Chest	24 (19.0)	31 (24.8)	55 (21.9)
	Lower limb	20 (15.9)	11 (8.8)	31 (12.4)
	Upper limb or shoulder	11 (8.7)	13 (10.4)	24 (9.6)
	Buttocks	11 (8.7)	7 (5.6)	18 (7.2)
	Head or neck	3 (2.4)	4 (3.2)	7 (2.8)
	Other	4 (3.2)	8 (6.4)	12 (4.8)
Complications	Yes	125 (99.2)	123 (98.4)	248 (98.8)
Concomitant drugs	Yes	126 (100.0)	125 (100.0)	251 (100.0)
Type of concomitant drug	Non-opioid analgesics	126 (100.0)	122 (97.6)	248 (98.8)
	NSAIDs	94 (74.6)	97 (77.6)	191 (76.1)
	Anti-cancer agents	84 (66.7)	89 (71.2)	173 (68.9)
	Adrenocorticosteroid	62 (49.2)	53 (42.4)	115 (45.8)
	Pregabalin	9 (7.1)	13 (10.4)	22 (8.8)
	Antidepressants	1 (0.8)	4 (3.2)	5 (2.0)
	Analgesic supportive therapy	0	1 (0.8)	1 (0.4)
Concomitant therapies	Yes	34 (27.0)	29 (23.2)	63 (25.1)

^a^Multiple types/sites were possible

Values are *n* (%) or mean ± SD, unless otherwise specified

*IR* immediate-release, *NSAID* non-steroidal anti-inflammatory drug, *SD* standard deviation, *VAS* visual analog scale (0–100 mm)

The primary endpoint could not be determined due to missing values at EOT/discontinuation for 2 patients in the bilayer tablet group and 5 patients in the IR capsule group. Therefore, the FAS comprised 244 patients (bilayer tablet group, 124; IR capsule group, 120).

Treatment adherence, which was assessed using the FAS, was high, with mean ± standard deviation (SD) medication compliance rates of 99.26% ± 2.44% in the bilayer tablet group and 99.15% ± 3.78% in the IR capsule group.

### VAS for pain at rest and during movement

**Fig. 3:**
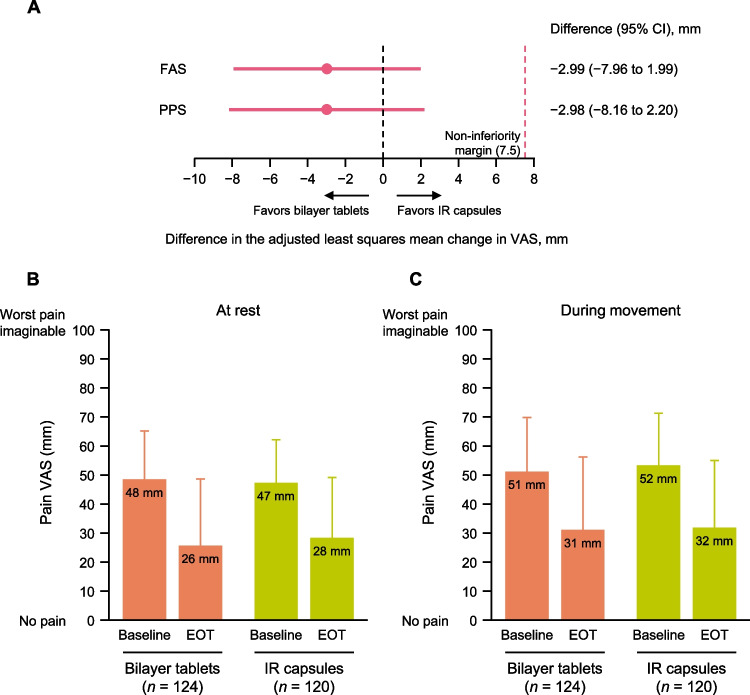
(A) Non-inferiority analysis of the adjusted mean change in VAS for pain at rest from baseline to EOT (primary endpoint). (B) Change in VAS for pain at rest from baseline to EOT. (C) Change in VAS for pain during movement from baseline to EOT. Values in B and C are mean ± standard deviation. *CI* confidence interval, *EOT* end of treatment, *FAS* full analysis set, *IR* immediate-release, *PPS* per-protocol set, *VAS* visual analog scale (100 mm)

**Fig. 4:**
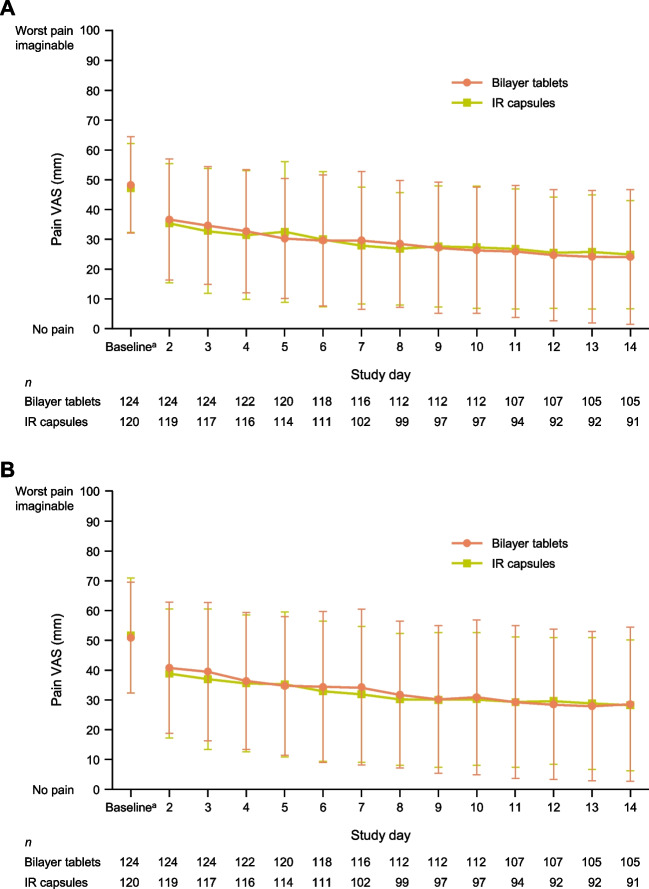
Changes in VAS over time for pain at rest (A) and pain during movement (B). Values are mean ± standard deviation. ^a^Averaged over 3 days before starting study drug administration. *IR* immediate-release, *VAS* visual analog scale (100 mm)

The adjusted mean change in VAS for pain at rest from baseline to EOT/discontinuation (FAS) was − 22.07 mm for the bilayer tablet group and − 19.08 mm for the IR capsule group, corresponding to a between-group adjusted mean difference of − 2.99 mm (95% CI − 7.96 to 1.99 mm). The upper 95% CI bound was less than the predefined non-inferiority margin of 7.5 mm, demonstrating non-inferiority of the bilayer tablets to the IR capsules (Fig. 3A). In the supplementary analysis using the PPS, the adjusted mean difference between the two groups was − 2.98 mm (95% CI − 8.16 to 2.20 mm), which was also less than the non-inferiority margin. Figure 3B shows the mean values for VAS for pain at rest at baseline and at EOT/discontinuation in both groups. Figure 3C shows the corresponding data for the VAS for pain during movement. The adjusted mean change in the VAS for pain during movement was − 20.43 and − 19.06 mm in the bilayer tablet and IR capsule groups, respectively, with an adjusted mean difference of − 1.38 mm (95% CI − 6.79 to 4.03 mm). The improvements in VAS scores for pain at rest and during movement on each day showed strong similarity in both groups (Fig. 4).

The proportion of patients with a clinically relevant improvement in pain at rest (at EOT/discontinuation) was numerically greater in the bilayer tablet group (87/124, 70.2%) than in the IR capsule group (69/120, 57.5%). Furthermore, a slightly greater proportion of patients in the bilayer tablet group experienced a clinically relevant improvement in pain during movement (71/124, 57.3% vs 60/120, 50.0%).

### Estimated total duration of pain per day

The estimated total duration of pain per day was assessed using a five-item scale on study days 2–14 of the treatment period. On study day 2, 50.0% (62/124) of patients in the bilayer tablet group and 54.6% (65/119) of patients in the IR capsule group reported that their duration of pain was < 4 h. This percentage increased slightly in both groups to 59.6% (62/104) in the bilayer tablet group and 60.4% (55/91) in the IR capsule group on study day 14 (Supplementary Table 2).

### Sleep

The majority of patients reported that their sleep was good during the treatment period. The percentage of patients who reported that they “slept well” or “slept moderately well” ranged from 77% to 86% in the bilayer tablet group and from 77% to 87% in the IR capsule group (Supplementary Fig. 1). The percentage of patients who reported that they “slept well” varied from 25% to 38% in the bilayer tablet group and from 24% to 41% in the IR capsule group.

### Use of rescue medications

Rescue medications (one or more doses of tramadol capsule) were used by 14.8%–22.3% of patients in the bilayer tablet group and by 10.3%–22.8% in the IR capsule group (Supplementary Fig. 2A, B). The frequency of rescue medication use remained broadly stable throughout the treatment period. The majority of patients who used rescue medication took a single dose in each group, with percentages that ranged from 9.8% to 18.5% in the bilayer tablet group and from 6.8% to 19.6% in the IR capsule group (Supplementary Fig. 2C, D).

### Quality of life

There were no marked changes in the quality of life (QOL) scores determined using the EuroQOL 5-dimension, 5-level questionnaire (EQ-5D-5L) (Supplementary Fig. 3**)**, or in the individual domains, during the treatment period in either group.

### Safety

#### Treatment period

During the 14-day treatment period, adverse events (AEs) were reported for 97 (77.0%) patients in the bilayer tablet group and 101 (80.8%) of patients in the IR capsule group (Table 2). This included severe AEs in 4.8% and 5.6% of patients, respectively, and serious AEs in 8.7% and 13.6% of patients, respectively. However, few of these AEs were thought to be related to the study drugs because most of the AEs corresponded to exacerbations of the primary or metastatic cancer. In the bilayer tablet group, one patient experienced a severe adverse drug reaction (ADR) and two patients experienced serious ADRs. No severe or serious ADRs were reported in the IR capsule group. AEs resulted in death in 4 (3.2%) patients in the bilayer tablet group and 3 (2.4%) patients in the IR capsule group, but none of these events were considered related to the study drugs. ADRs resulted in discontinuation of the study drug for 10 (7.9%) patients in the bilayer tablet group and 11 (8.8%) patients in the IR capsule group. No ADRs resulted in a reduction in the doses of the study drugs. The three most common AEs in both treatment groups were nausea, constipation, and vomiting (Table 2). The frequencies and types of ADRs were generally similar between the two treatment groups (Supplementary Table 3). ADRs that occurred in ≥ 2% of patients in the bilayer tablet group were nausea (bilayer tablet group and IR capsule group: 27.8% and 32.0%), constipation (19.8% and 16.0%), vomiting (16.7% and 16.8%), somnolence (14.3% and 9.6%), dizziness (7.1% and 4.8%), decreased appetite (6.3% and 0.8%), and malaise (2.4% and 0.8%). There were no consistent trends or notable findings regarding vital signs or 12-lead electrocardiography.

**Table 2:** Summary of AEs and ADRs during the treatment period

	Bilayer tablets	IR capsules
*N*	126	125
AEs	97 (77.0)	101 (80.8)
Severe AEs	6 (4.8)	7 (5.6)
AEs resulting in death	4 (3.2)	3 (2.4)
Serious AEs	11 (8.7)	17 (13.6)
AEs resulting in discontinuation of the study drug	15 (11.9)	25 (20.0)
AEs leading to dose reduction of the study drug	1 (0.8)	0
ADRs	74 (58.7)	67 (53.6)
Severe ADRs	1 (0.8)	0
ADRs resulting in death	0	0
Serious ADRs	2 (1.6)	0
ADRs resulting in discontinuation of the study drug	10 (7.9)	11 (8.8)
ADRs leading to dose reduction of the study drug	0	0
AEs in ≥ 2% of patients during the treatment period, by preferred term (MedDRA/J version 23.0)^a^		
Nausea	41 (32.5)	44 (35.2)
Constipation	28 (22.2)	21 (16.8)
Vomiting	26 (20.6)	26 (20.8)
Somnolence	18 (14.3)	12 (9.6)
Decreased appetite	16 (12.7)	5 (4.0)
Dizziness	10 (7.9)	6 (4.8)
Malaise	7 (5.6)	2 (1.6)
Diarrhea	6 (4.8)	5 (4.0)
Neutrophil count decreased	4 (3.2)	2 (1.6)
Stomatitis	3 (2.4)	2 (1.6)
White blood cell count decreased	3 (2.4)	3 (2.4)
Gastric cancer	3 (2.4)	1 (0.8)
Pruritus	2 (1.6)	3 (2.4)
Pancreatic carcinoma	1 (0.8)	3 (2.4)
Oropharyngeal pain	1 (0.8)	3 (2.4)
Edema peripheral	0	3 (2.4)
Metastases to liver	0	3 (2.4)
Fall	0	3 (2.4)
Bone marrow failure	0	3 (2.4)

Values are *n* (%) of patients

^a^Ordered by descending frequency in the bilayer tablet group

*ADR* adverse drug reaction, *AE* adverse event, *IR* immediate-release

#### Follow-up period

During the follow-up period, AEs were reported for 43 (34.1%) patients in the bilayer tablet group and 46 (36.8%) patients in the IR capsule group, indicating no difference in safety during this period (Supplementary Table 4). ADRs were reported for 3 (2.4%) patients in the bilayer tablet group and 2 (1.6%) patients in the IR capsule group. One AE resulted in death in the bilayer tablet group, but the event was not considered related to the study drug. Severe and serious AEs were reported in both groups, but were not considered related to the study drugs. The most frequent AEs during the follow-up period were constipation, nausea, and vomiting in the bilayer tablet group and nausea, decreased appetite, and constipation in the IR capsule group. There were no reported cases of drug dependency based on the standardized MedDRA query “Drug abuse and dependence.”

## Discussion

Our aim was to investigate the non-inferiority of a bilayer tablet formulation of tramadol, comprising IR and SR layers, versus an IR capsule formulation in terms of managing cancer pain. The two treatments achieved similar improvements in the VAS for pain at rest, satisfying the criterion for non-inferiority, which was confirmed in the PPS analysis. Additionally, the changes in VAS for pain at rest and during movement on each study day, percentages of patients who slept well or moderately well, use of rescue medication, and EQ-5D-5L QOL scores were highly comparable, indicating highly similar effects of both formulations on pain control. The improvement in pain was rapid, from within 2 days of starting administration, and showed good stability throughout the study in both groups. Overall, these findings indicate that twice-daily administration of the bilayer tablets is as effective as four-times-daily IR tramadol for managing cancer pain.

Opioids are frequently used to manage cancer pain [15–21], due to their effectiveness and inclusion in clinical guidelines/recommendations [4–8]. Furthermore, studies have shown that opioids can improve QOL by alleviating cancer-related pain [22–27]. Here, we have shown that two formulations of tramadol can achieve a clinically relevant improvement in cancer pain at rest and during movement, and both formulations were comparable in terms of other outcomes, including sleep quality, use of rescue medications, and QOL. Therefore, our findings provide further support for using tramadol to manage cancer pain, and that physicians could choose an administration regimen (e.g., twice-daily or four-times-daily) that might be most suitable for the individual patient.

We also investigated the safety of both study drugs in terms of AEs/ADRs during the 14-day treatment period and 7-day follow-up period. During the treatment period, AEs were reported for 77.0% and 80.8% of patients in the bilayer tablet and IR capsule groups, respectively, while ADRs were reported for 58.7% and 53.6%, respectively. These values seem reasonable when we consider the frequencies of AEs reported in the initial open-label treatment escalation periods (80.6% and 78.7% in the knee osteoarthritis and postherpetic neuralgia studies, respectively) of two previous dose-withdrawal studies using the bilayer tablet formulation [11, 12]. We enrolled opioid-naïve patients, which may increase the risk of opioid-related AEs and ADRs. Additionally, all of the patients were using concomitant drugs, such as non-opioid analgesics, two-thirds were receiving anti-cancer therapies, and nearly half were using a corticosteroid. Thus, the frequencies of AEs/ADRs are within expected ranges. The most common types of AEs and ADRs were nausea, constipation, vomiting, and somnolence, which are known to be associated with tramadol [5, 6, 28]. Nevertheless, there were few moderate or severe ADRs that were likely to interfere with daily activities, and only two serious ADRs and one severe ADR were reported. Overall, physicians should take appropriate care when prescribing tramadol while monitoring its safety, especially in opioid-naïve patients.

Clinical guidelines position opioids, including tramadol, as options for managing cancer pain [4–8]. If acetaminophen or NSAIDs do not provide sufficient pain control, it may be possible to switch to these bilayer tramadol tablets, which have already shown good long-term efficacy and tolerability in patients with chronic non-cancer pain [10–12]. These bilayer tablets could be started early in the patient’s clinical course and stepped down when no longer required, in accordance with WHO recommendations for the initiation, maintenance, and cessation of opioids [4].

Several preparations of tramadol, including IR and SR formulations, have been developed and are used to manage cancer pain. However, there are some potential disadvantages of the available formulations related to their pharmacokinetic properties. In particular, the pharmacokinetics of once-daily SR formulations may not be sufficient to maintain effective pain relief over the 24-h period between each dose [29]. As such, patients may require frequent use of rescue medications to maintain adequate pain relief. By comparison, the pharmacokinetics of IR formulations may provide adequate efficacy, but the frequent administration (four-times-daily) may pose a pill burden, which was associated with decreased treatment satisfaction and reduced medication adherence in other settings [30–34]. Further, studies in other settings suggested that patients were less adherent to a four-times-daily regimen than a twice-daily regimen [35–37]. Thus, patients may show better adherence to a twice-daily regimen, especially one that provides a rapid onset of action through the IR component and prolonged action through the SR component. Accordingly, we hypothesize that these bilayer tramadol tablets could offer greater compliance and at least comparable effectiveness to alternative tramadol regimens requiring more frequent administration.

### Limitations

There are some limitations of this study that warrant mention. In particular, the treatment period was relatively short (14 days), which prevented us from assessing the longer-term effectiveness of tramadol. This period was selected based on an earlier study in Japan of the same length [14] and in consideration of the potential impact of anti-cancer therapy in longer-term studies. We should also consider the possibility that safety assessments may have been influenced by the use of concomitant drugs, including anti-cancer therapies, that might have inflated the frequency of AEs in this study. However, this risk seems low because the types of AEs were generally consistent with the known safety profile of tramadol. Because the study lacked a placebo group, we cannot exclude the possibility of a placebo or trial effect. However, this was deemed unethical because the patients all reported clinically significant pain despite treatment with non-opioid analgesics that would have necessitated other treatments or high rates of rescue medication. Furthermore, a placebo group was deemed unnecessary because both study drugs had already been evaluated in placebo-controlled trials of other indications [11, 12, 38, 39]. We did not use a cross-over design, which may have been useful to evaluate whether patients had preferences regarding formulation and administration frequency.

### Conclusions

Twice-daily administration of bilayer tramadol tablets comprising 35% immediate-release and 65% sustained-release tramadol was as effective as four-times-daily IR capsules regarding the improvement in the VAS for pain at rest. We also observed strong similarity in the other effectiveness outcomes, including the improvements in VAS for pain at rest and during movement on each study day, sleep quality, use of rescue medications, and EQ-5D-5L QOL scores. Furthermore, the safety profiles of both study groups were consistent with the known safety profile for tramadol. Overall, these findings indicate that bilayer tramadol tablets are an effective and tolerable treatment option for managing cancer pain, comparable to four-times-daily administration of IR capsules.

### Supplementary Information

Below is the link to the electronic supplementary material.

Supplementary file1 (PDF 421 KB)

## Data Availability

The datasets generated and/or analyzed during the current study are available from the corresponding author on reasonable request.
