# Supplementary material for: Phase III study of bilayer sustained-release tramadol tablets in patients with cancer pain: a double-blind parallel-group, non-inferiority study with immediate-release tramadol capsules as an active comparator
Source: Support Care Cancer. 2023 Dec 29;32(1):69. doi: 10.1007/s00520-023-08242-z (PMC10756890; doi:10.1007/s00520-023-08242-z)
Supplement: Supplementary file 1 — Supplementary file1 (PDF 421 KB) [file 520_2023_8242_MOESM1_ESM.pdf]

SUPPORTING INFORMATION

Supplementary Methods

Major exclusion criteria

Major exclusion criteria were symptoms associated with brain tumor/metastasis/encephalopathy/cerebral infarction/cerebral hemorrhage, comorbid (or history) of convulsive seizure or epileptic seizure, serious systemic disorders, mental disorders, hypersensitivity to tramadol or other opioids, history of drug/alcohol dependency/abuse, other type of pain likely to interfere with the study, recent treatment (surgery, radiotherapy, nerve block or stimulation-produced analgesia) within 7 days before the study or planned during the study, recent use of a monoamine oxidase inhibitor within 14 days before the study, recent start or expected change in chemotherapy that would interfere with the study or make it difficult to evaluate efficacy and safety, participation in a clinical study up to 28 days before the start of study drug administration, women who were pregnant (including suspected or planning pregnancy) or breastfeeding, and patients who the investigator deemed ineligible for the study.

Approved and prohibited therapies

Other than the study drugs and approved use of rescue medication, patients were permitted to take non-steroidal anti-inflammatory drugs (NSAIDs) and acetaminophen (paracetamol) providing their dose and administration frequency remained unchanged from the date of consent until the end of treatment or discontinuation. Temporary administration of NSAIDs or acetaminophen was also permitted to manage fever and inflammation. Other drugs (analgesic adjuvants, corticosteroids, non-opioid analgesics other than NSAIDs, and acetaminophen) could also be used during the study providing their dose and administration frequency remained unchanged during the study.

Drugs prohibited during the study included other opioids, monoamine oxidase inhibitors, central nervous system inhibitors, tricyclic antidepressants, serotonin agonists, linezolid, carbamazepine, quinidine,

digoxin, warfarin, ondansetron, and other investigational drugs.

Antineoplastic drugs could be continued at the same dose and frequency of administration from before enrolment. Changes to antineoplastic drugs were not permitted during the trial; if changes to antineoplastic drugs were expected or deemed necessary during the trial, the patient was to discontinue the study to prioritize cancer care. However, anti-cancer surgery or surgery requiring general anesthesia, radiotherapy, nerve blocks/stimulant analgesia, and medical device trials were prohibited.

Dosing of study drugs and rescue medication

The dosing of each formulation started at 50 mg/day in the evening on the first day of treatment (study day 1), followed by 100 mg/day on study day 2. On study days 2–10, the tramadol dose could be escalated by 100 mg/day if analgesia was determined to be inadequate based on the use of rescue medication on the previous day and if the investigator/subinvestigator considered escalation would be tolerated by the patient. The tramadol dose could not be escalated between study days 11 and 14, but rescue medication was permitted on those days. If analgesia was considered to be inadequate at 300 mg/day, the treatment was to be discontinued. Dose reductions of up to 100 mg/day were permitted if deemed necessary by the investigator/subinvestigator (e.g., following an adverse event [AE]).

As rescue pain medication, patients were permitted to take one dose of immediate-release (IR) tramadol between the fixed interval doses with an interval of ≥1 h between the dose of rescue medication and the allocated study drug. One capsule per dose was permitted for patients using the study drug at doses of 100 or 200 mg/day (up to four doses per day) and two capsules per dose was permitted for patients using the study drug at a dose of 300 mg/day (up to two doses per day). However, rescue medication was not permitted for patients aged ≥75 years at the time of consent if they were using the study drug at a dose of 300 mg/day.

Planned study duration

The duration of the treatment period was chosen based on the results of an earlier Phase III study of tramadol IR capsules performed in Japan, in which the mean change in visual analog scale (VAS) for pain was −28.9 mm over a 14-day treatment period [1]. Furthermore, a longer treatment period was deemed unsuitable because of the possibility that anti-cancer therapies may interfere with the study conduct, necessitate discontinuation, or influence the interpretation of the results.

Secondary endpoints and safety assessments

Patients completed diaries each day to record their administration of the study drug, the use of rescue medications, the estimated duration of pain per day, and sleep at night. The estimated duration of pain was recorded using a five-item scale: <4 h, 4 to <8 h, 8 to <12 h, ≥12 h, and all day. Sleep was rated on a four-point scale: "slept well," "slept moderately well," "did not sleep well," and "did not sleep at all." Quality of life (QOL) was assessed using the EuroQOL 5-dimension, 5-level questionnaire (EQ-5D-5L) at visits 2, 3, and 4. Vital signs and 12-lead electrocardiography were assessed using standard methods.

AEs were to be reported throughout the study and were coded according to MedDRA/J ver. 23.0. The causal relationship between AEs and the study drug was determined based on the timing of administration of antineoplastic drugs and tramadol, as well as the known safety profile of each drug used by the patient. No dependency survey was conducted, but AEs associated with withdrawal symptoms were collected and assessed with reference to the standardized MedDRA query (SMQ) "Drug abuse and dependence."

Sample size calculation

The sample size was determined with reference to a previous Phase III study of tramadol IR capsules performed in Japan [1]. In that study of 48 patients treated with tramadol for 2 weeks (baseline VAS ≥25 mm), the mean change in VAS was −28.9 mm (standard deviation 17.0 mm) at completion/discontinuation. Therefore, we assumed a change in VAS of −28.9 mm (standard deviation

17.0 mm) between visits 2 and 4 (or discontinuation) in the bilayer tablet and IR capsule groups in this study. Assuming a clinically meaningful VAS difference of 15 mm and a non-inferiority margin of 7.5 mm, the number of subjects needed to verify non-inferiority was 109 per group at a significance level of 2.5% (one-sided), and a power of 90%. Considering the potential number of dropouts, we planned to enroll 120 patients per group.

Statistical analyses

The change in the VAS for pain during movement was analyzed by analysis of covariance in a similar manner to the primary endpoint, albeit without testing non-inferiority. We also used a mixed model for repeated measures to assess the changes in VAS scores at rest and during movement on each study day during the treatment period without imputing missing values. In these analyses, the baseline score was included as a covariate, and treatment group, study day, and an interaction between treatment group and study day were included as fixed effects.

Descriptive analyses were performed for the other efficacy and safety outcomes, including percentages of patients with a clinically relevant improvement in VAS scores at rest and during movement, frequencies of rescue medication, duration of pain, sleep at night, EQ-5D-5L QOL scores, frequencies of AEs/adverse drug reactions, and vital signs.

Reference

1. Nippon Shinyaku Co., Ltd. Summary of individual studies. Attachments to the application for marketing approval of Tramal Capsules. Available at: https://www.pmda.go.jp/drugs/2010/P201000036/index.html. Accessed July 6, 2023 (in Japanese)

Supplementary Tables

Supplementary Table 1 Definition of clinically relevant improvement in pain based on the pain VAS recorded at baseline and after starting study drug administration

**Baseline VAS (mm)** [a]	**Pain VAS (mm) during study drug administration** [b]										
	**0–4**	**5–14**	**15–24**	**25–34**	**35–44**	**45–54**	**55–64**	**65–74**	**75–84**	**85–94**	**95–100**
**25–34**	1	2	2	3	4	4	5	5	5	5	5
**35–44**	1	2	2	3	4	4	5	5	5	5	5
**45–54**	1	1	2	2	3	4	4	5	5	5	5
**55–64**	1	1	2	2	3	4	4	5	5	5	5
**65–74**	1	1	1	2	2	3	4	4	5	5	5
**75–84**	1	1	1	2	2	3	4	4	5	5	5
**85–94**	1	1	1	1	2	2	3	4	4	5	5
**95–100**	1	1	1	1	2	2	3	4	4	5	5

[a] Averaged over 3 days before starting study drug administration. Patients with VAS <25 mm were excluded from the study.

[b] Averaged over 3 days before visits 3 or 4.

1 = marked improvement; 2 = moderate improvement; 3 = mild improvement; 4 = unchanged; 5 = deteriorated

Clinically relevant improvement in pain was defined as marked improvement (1) or moderate improvement (2), as indicated by the shaded cells.

Modified from Hiraga K, Ohkuma S, Asano H, Honmura T, Nishimura T, Takeda F (2010) A Phase III clinical trial of NS-315 (tramadol hydrochloride), a weak opioid analgesic, in patients with cancer pain − randomized, double-blind, parallel, comparative study with morphine. Clin Med 26:569–584 (in Japanese).

Supplementary Table 2 Self-reported duration of pain per day

Study day	Duration of pain per day	Bilayer tablets (N = 124)	IR capsules (N = 120)
2	N	124	119
	<4 h	62 (50.0)	65 (54.6)
	≥4 to <8 h	32 (25.8)	29 (24.4)
	≥8 to <12 h	8 (6.5)	12 (10.1)
	≥12 h	17 (13.7)	8 (6.7)
	All day	5 (4.0)	5 (4.2)
3	N	124	116
	<4 h	60 (48.4)	63 (54.3)
	≥4 to <8 h	33 (26.6)	30 (25.9)
	≥8 to <12 h	14 (11.3)	10 (8.6)
	≥12 h	15 (12.1)	10 (8.6)
	All day	2 (1.6)	3 (2.6)
4	N	122	116
	<4 h	67 (54.9)	62 (53.4)
	≥4 to <8 h	26 (21.3)	32 (27.6)
	≥8 to <12 h	15 (12.3)	14 (12.1)
	≥12 h	14 (11.5)	5 (4.3)
	All day	0	3 (2.6)
5	N	120	114
	<4 h	65 (54.2)	69 (60.5)
	≥4 to <8 h	35 (29.2)	20 (17.5)
	≥8 to <12 h	7 (5.8)	17 (14.9)
	≥12 h	12 (10.0)	5 (4.4)
	All day	1 (0.8)	3 (2.6)
6	N	117	111
	<4 h	65 (55.6)	66 (59.5)
	≥4 to <8 h	31 (26.5)	27 (24.3)
	≥8 to <12 h	10 (8.5)	11 (9.9)
	≥12 h	11 (9.4)	5 (4.5)
	All day	0	2 (1.8)
7	N	115	101
	<4 h	64 (55.7)	63 (62.4)

	≥4 to <8 h	30 (26.1)	25 (24.8)
	≥8 to <12 h	9 (7.8)	8 (7.9)
	≥12 h	11 (9.6)	4 (4.0)
	All day	1 (0.9)	1 (1.0)
8	*N*	112	98
	<4 h	71 (63.4)	66 (67.3)
	≥4 to <8 h	22 (19.6)	17 (17.3)
	≥8 to <12 h	9 (8.0)	10 (10.2)
	≥12 h	10 (8.9)	4 (4.1)
	All day	0	1 (1.0)
9	*N*	112	97
	<4 h	73 (65.2)	63 (64.9)
	≥4 to <8 h	21 (18.8)	19 (19.6)
	≥8 to <12 h	12 (10.7)	8 (8.2)
	≥12 h	6 (5.4)	6 (6.2)
	All day	0	1 (1.0)
10	*N*	112	97
	<4 h	70 (62.5)	55 (56.7)
	≥4 to <8 h	27 (24.1)	26 (26.8)
	≥8 to <12 h	11 (9.8)	10 (10.3)
	≥12 h	4 (3.6)	4 (4.1)
	All day	0	2 (2.1)
11	*N*	107	94
	<4 h	66 (61.7)	58 (61.7)
	≥4 to <8 h	25 (23.4)	24 (25.5)
	≥8 to <12 h	9 (8.4)	7 (7.4)
	≥12 h	7 (6.5)	3 (3.2)
	All day	0	2 (2.1)
12	*N*	107	92
	<4 h	66 (61.7)	54 (58.7)
	≥4 to <8 h	30 (28.0)	25 (27.2)
	≥8 to <12 h	4 (3.7)	8 (8.7)
	≥12 h	7 (6.5)	4 (4.3)
	All day	0	1 (1.1)
13	*N*	105	92
	<4 h	67 (63.8)	56 (60.9)

	≥4 to <8 h	25 (23.8)	21 (22.8)
	≥8 to <12 h	7 (6.7)	10 (10.9)
	≥12 h	6 (5.7)	4 (4.3)
	All day	0	1 (1.1)
14	*N*	104	91
	<4 h	62 (59.6)	55 (60.4)
	≥4 to <8 h	27 (26.0)	25 (27.5)
	≥8 to <12 h	11 (10.6)	5 (5.5)
	≥12 h	4 (3.8)	5 (5.5)
	All day	0	1 (1.1)

Values are *n* (%) of available patients.

IR immediate-release

Supplementary Table 3 Adverse drug reactions

	Bilayer tablets	IR capsules
N	126	125
Any ADR	74 (58.7)	67 (53.6)
ADRs during the treatment period, by preferred term (MedDRA/J version 23.0)[a]		
Nausea	35 (27.8)	40 (32.0)
Constipation	25 (19.8)	20 (16.0)
Vomiting	21 (16.7)	21 (16.8)
Somnolence	18 (14.3)	12 (9.6)
Dizziness	9 (7.1)	6 (4.8)
Decreased appetite	8 (6.3)	1 (0.8)
Malaise	3 (2.4)	1 (0.8)
Thirst	2 (1.6)	1 (0.8)
Blood creatine phosphokinase increased	2 (1.6)	1 (0.8)
Liver function test increased	1 (0.8)	1 (0.8)
Pruritus	1 (0.8)	1 (0.8)
Hypoesthesia	1 (0.8)	0
Headache	1 (0.8)	0
Feeling abnormal	1 (0.8)	0
Neutrophil count increased	1 (0.8)	0
Monocyte count increased	1 (0.8)	0
Night sweats	1 (0.8)	0
Urticaria	1 (0.8)	0
Tinnitus	1 (0.8)	0
Feces hard	0	1 (0.8)
Taste disorder	0	1 (0.8)
Dehydration	0	1 (0.8)
Platelet count decreased	0	1 (0.8)
White blood cell count decreased	0	1 (0.8)
Vertigo	0	1 (0.8)
Restlessness	0	1 (0.8)
Dysuria	0	1 (0.8)

Values are *n* (%) of patients.

[a]Ordered by descending frequency in the bilayer tablet group.

ADR adverse drug reaction, *IR* immediate-release

Supplementary Table 4 Summary of AEs and ADRs during the follow-up period

	Bilayer tablets	IR capsules
N	126	125
AEs	43 (34.1)	46 (36.8)
Severe AEs	5 (4.0)	4 (3.2)
AEs resulting in death	1 (0.8)	0
Serious AEs	8 (6.3)	7 (5.6)
AEs resulting in discontinuation of the study drug	0	0
AEs leading to dose reduction of the study drug	0	0
ADRs	3 (2.4)	2 (1.6)
Severe ADRs	0	0
ADRs resulting in death	0	0
Serious ADRs	0	0
ADRs resulting in discontinuation of the study drug	0	0
ADRs leading to dose reduction of the study drug	0	0
AEs in ≥2% of patients during the follow-up period, by preferred term (MedDRA/J version 23.0)[a]		
Constipation	6 (4.8)	9 (7.2)
Nausea	5 (4.0)	13 (10.4)
Vomiting	4 (3.2)	5 (4.0)
Ascites	3 (2.4)	0
Decreased appetite	2 (1.6)	10 (8.0)
Somnolence	1 (0.8)	6 (4.8)
Malaise	1 (0.8)	4 (3.2)
Diarrhea	1 (0.8)	3 (2.4)
Insomnia	1 (0.8)	3 (2.4)
Hypoglycemia	0	3 (2.4)
Procedural pain	0	3 (2.4)

Values are *n* (%) of patients.

[a]Ordered by descending frequency in the bilayer tablet group.

ADR adverse drug reaction, *AE* adverse event, *IR* immediate-release

Supplementary Figures

Supplementary Fig. 1 Changes in the quality of nocturnal sleep over time

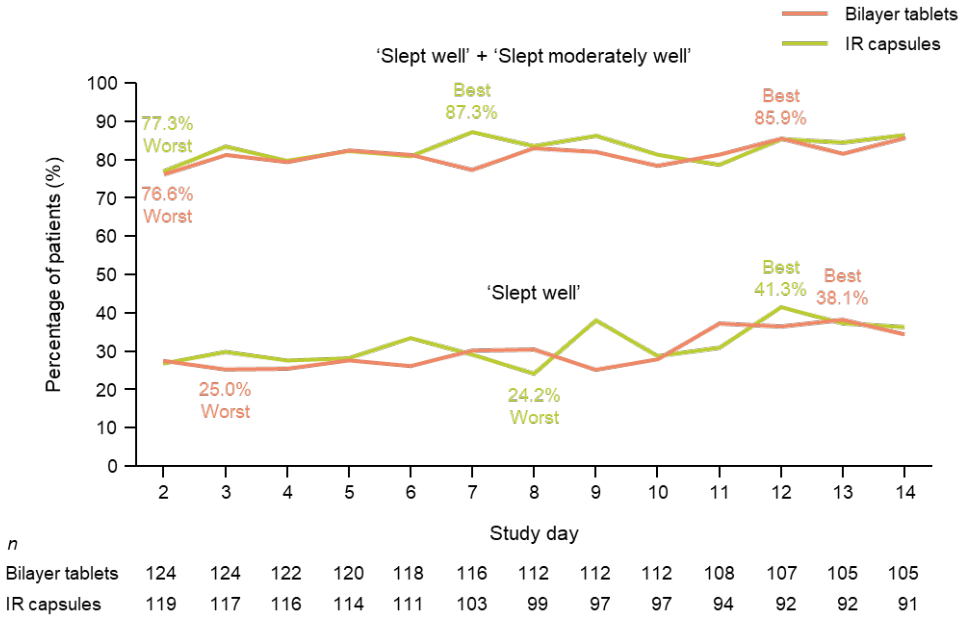

The quality of sleep was assessed using a four-point scale: "slept well," "slept moderately well," "did not sleep well," or "did not sleep at all." The upper lines show the percentages of patients who reported that they "slept well" or "slept moderately well." The lower lines show the percentages of patients who reported that they "slept well."

IR immediate-release

Supplementary Fig. 2 Use of rescue medication during the treatment period

(A,B) Percentages of patients who used any or no rescue medication on each study day in the bilayer tablet group (A) and IR capsule group (B).

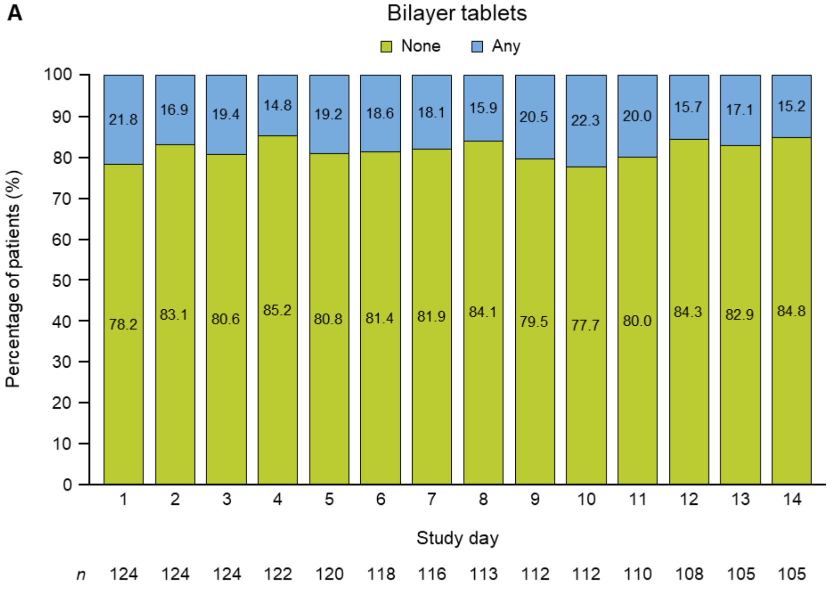

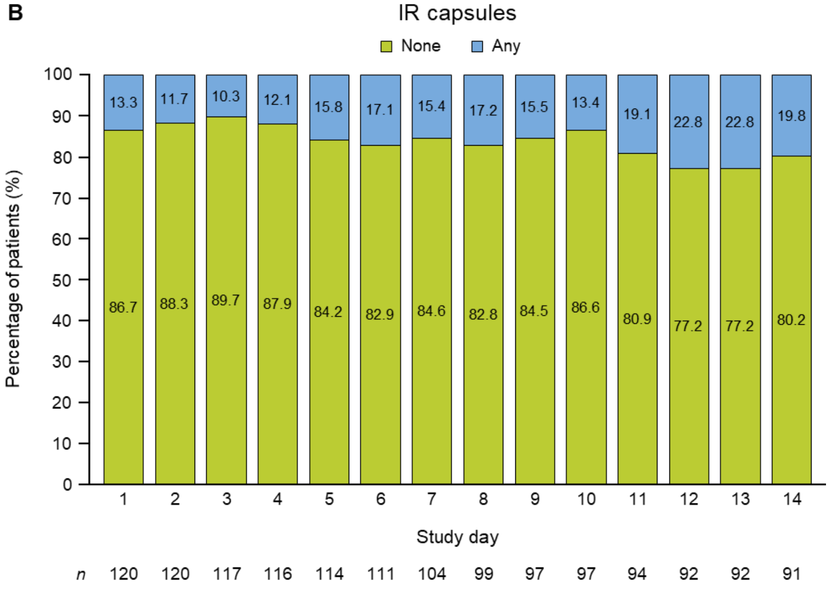

(C,D) Percentages of patients who used one, two, three, or four doses of rescue medication on each day in the bilayer tablet group (C) and IR capsule group (D).

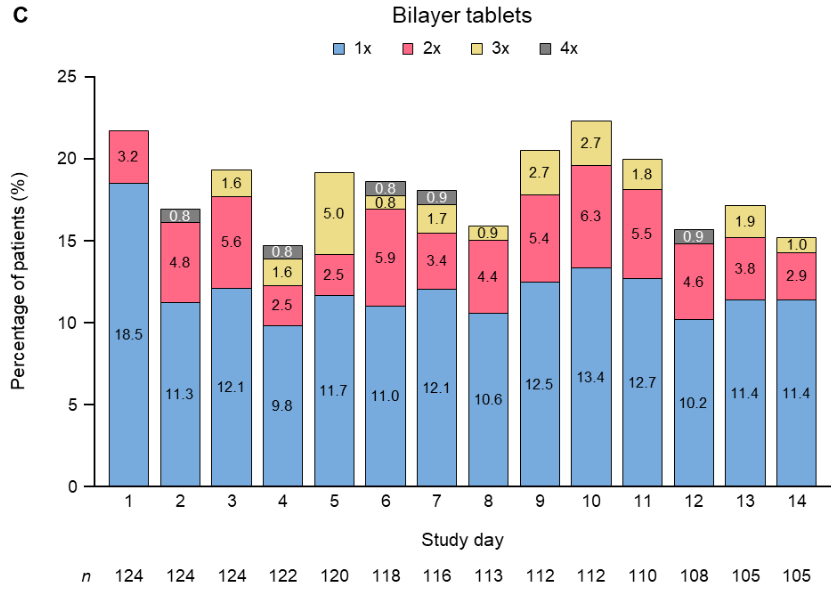

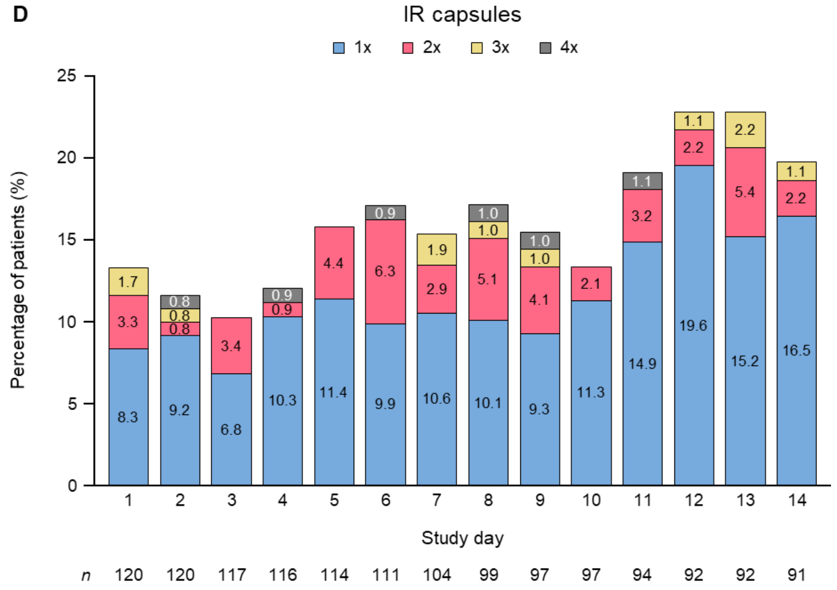

Rescue medication on study day 1 comprised the period from the start of study drug administration (evening on study day 1) to the next morning on study day 2. For subsequent study days, rescue medications were counted through to the following morning.

IR immediate-release

Supplementary Fig. 3 EQ-5D-5L QOL scores during the treatment period

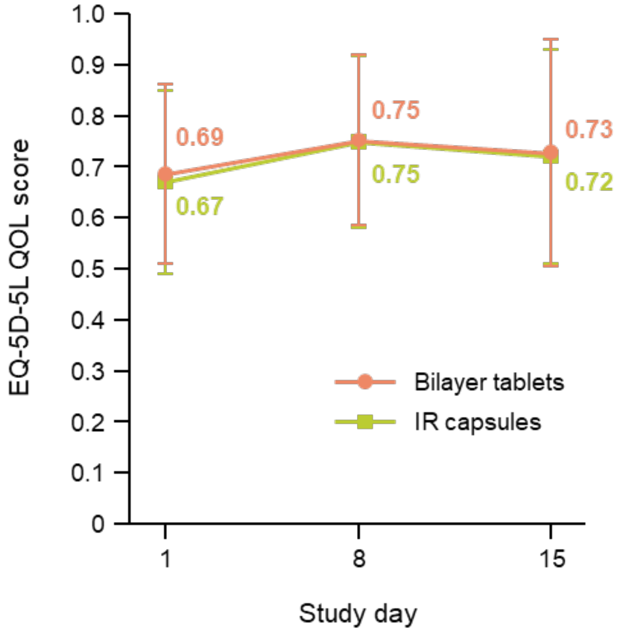

n			
Bilayer tablet	124	113	122
IR capsule	120	100	115

Study day 15 includes the score at the end of treatment or at discontinuation.

Values are mean ± standard deviation.

EQ-5D-5L QOL EuroQOL 5-dimension, 5-level quality of life questionnaire, *IR* immediate-release
